# Developing an Ear Prosthesis Fabricated in Polyvinylidene Fluoride by a 3D Printer with Sensory Intrinsic Properties of Pressure and Temperature

**DOI:** 10.3390/s16030332

**Published:** 2016-03-04

**Authors:** Ernesto Suaste-Gómez, Grissel Rodríguez-Roldán, Héctor Reyes-Cruz, Omar Terán-Jiménez

**Affiliations:** Department of Electrical Engineering, Section of Bioelectronics, Center for Research and Advanced Studies, CINVESTAV-IPN, Av. IPN 2508, Col. San Pedro Zacatenco, C.P. 07360, D.F., Mexico; grodriguezr@cinvestav.mx (G.R.-R.); van1321@hotmail.com (H.R.-C.); omartjmz@hotmail.com (O.T.-J.)

**Keywords:** 3D printer, pressure, prostheses, PVDF, smart materials, temperature

## Abstract

An ear prosthesis was designed in 3D computer graphics software and fabricated using a 3D printing process of polyvinylidene fluoride (PVDF) for use as a hearing aid. In addition, the prosthesis response to pressure and temperature was observed. Pyroelectric and piezoelectric properties of this ear prosthesis were investigated using an astable multivibrator circuit, as changes in PVDF permittivity were observed according to variations of pressure and temperature. The results show that this prosthesis is reliable for use under different conditions of pressure (0 Pa to 16,350 Pa) and temperature (2 °C to 90 °C). The experimental results show an almost linear and inversely proportional behavior between the stimuli of pressure and temperature with the frequency response. This 3D-printed ear prosthesis is a promising tool and has a great potentiality in the biomedical engineering field because of its ability to generate an electrical potential proportional to pressure and temperature, and it is the first time that such a device has been processed by the additive manufacturing process (3D printing). More work needs to be carried out to improve the performance, such as electrical stimulation of the nervous system, thereby extending the purpose of a prosthesis to the area of sensory perception.

## 1. Introduction

Recent advances in bionics and prosthetics have combined different techniques to develop, in the last few years, aesthetic and functional prostheses, which allow people with physical disabilities caused either by an accident or a genetic deformity to get on with their mode of life. Ear prostheses (which in common parlance are called “artificial ears”) are an example of progress in this area. They are common used in patients with microtia, which is a congenital malformation characterized by the underdevelopment of an ear or two. In addition to the psychological effect that can cause a malformed ear and usually, it is divided into several classifications that described changes of shape or deformations in the ear structures, like a small ear, partially-formed ear, the absence of the external ear (pinna) and the absence of the total ear (anotia). Thus, subjects with microtia have moderate to severe hearing loss; it is critical for humans to have good audition in order to have normal speech and bilateral hearing to detect directionality, so ear prostheses have become the most recommendable alternative, allowing the patient to recover both the functionality and appearance of a natural ear [1].

This paper will present the results, including the stages of the design, fabrication and characterization of a 3D-printed ear prosthesis using polyvinylidene fluoride **(**PVDF), a polymeric smart material, which is used as either a sensor or a transducer due to its high piezoelectric, pyroelectric, photo-pyroelectric and ferroelectric properties [2,3,4,5,6].

PVDF is the most significant and studied polymer by Andrew J. Lovinger [7]; PVDF responds to pressure and electric (piezoelectric effect), temperature, sound, light and even moisture [8]. Other polymeric materials, which are not pure and exhibit these properties, are ZnO/PVDF with graphene for pressure and temperature sensing, described by James S. Lee *et al.* [5].

Due to PVDF being a ferroelectric smart material with piezoelectric and pyroelectric properties, the prosthesis was subjected to variations of pressure and temperature (between 0 and 16,350 Pa and 5 °C and 90 °C, respectively). The environment temperature of human living can be roughly from −40 to 50 °C, a range that is within the working temperature range of the PVDF ear prosthesis. In this context, as reported by the manufacturers, the lowest operating temperature of PVDF is −35 °C. Thus, the response of the mentioned stimuli is presented. This way, it is expected to restore sensory functions, giving the user the ability to perceive heat, cold, touch and pressure. 

## 2. Experimental Section

### 2.1. Design and Manufacturing

An ear prosthesis was created according to anthropometric parameters. It was designed in Blender 2.76, which is a 3D computer graphics software, and the file was exported to an stereolithography (STL) file. The prosthesis was printed in PVDF ((C_2_H_2_F_2_)_n_), which is a smart material that exhibits piezoelectric and pyroelectric properties and is manufactured with a 3D touch printer from Bits from Bytes with a *Z*-axis resolution of 0.125 mm (0.005”/125 microns); the diameter of the nozzle is 0.3 mm, and the technology used was fused deposition modeling (FDM), which processes the STL format, thus controlling the movements of the nozzle (horizontal and vertical directions). The nozzle is heated to melt the material, and the prosthesis is created by the extrusion of the PVDF filament in order to form thin layers, which cool partially, drawing a cross-section of the ear prosthesis onto the build plate.

The printing parameters used for the ear prosthesis are displayed in Table 1.

The PVDF used was pure and bought in rods with 3 mm of diameter, from Goodfellow Limited Cambridge (FV307910, Ermine Business Park, Huntingdon, UK); this PVDF is subjected to strict quality control to assure repeatability in its ferroelectric properties, *i.e*., the piezoelectric and pyroelectric properties of the printed ear are not affected.

### 2.2. Polarization

PVDF is polymorphic and has a crystalline phase (ß-phase). The ß-phase is the most relevant phase for practical ferroelectric, piezoelectric and pyroelectric applications. PVDF belongs to a non-centrosymmetric class, which is responsible for its piezoelectric properties. The ear prosthesis made of piezoelectric PVDF is preferred, because the processes commonly used to obtain piezoelectric and pyroelectric properties of PVDF in the ß-phase are achieved by applying a high voltage of at least 0.5 MV/cm, or by using plasma or corona poling [7].

The ear prosthesis of PVDF was poled using corona poling where a charge is injected applying a high voltage (15 kV) into the polymeric ear prosthesis. The prosthesis was placed on a copper plate, and a needle was situated over it at a distance of one centimeter. Besides, in corona poling, no electrodes are required as the poling process is done by a strong electrical field [7,9].

### 2.3. Characterization

**Figure 1:**
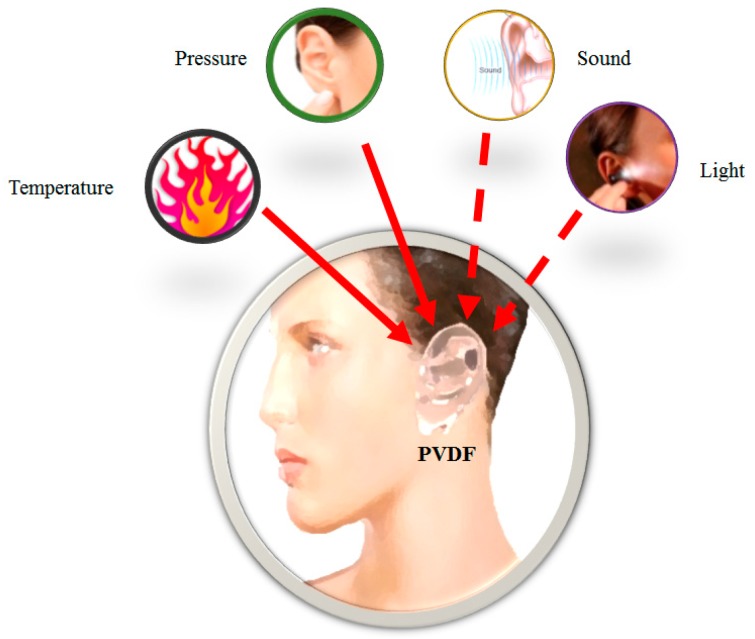
Diagram of the different stimuli applied to the PVDF prosthesis.

In this stage, the ear prosthesis was characterized by means of applying different stimuli, such as pressure, heat and cold (imitating human skin receptors), in order to register different responses of the PVDF prosthesis as a multisensory unit. Figure 1 shows the general diagram of the experiments that are mentioned above. 

### 2.4. Characterization of the Ferroelectric Properties of PVDF

#### 2.4.1. Hysteresis Loop

**Figure 2:**
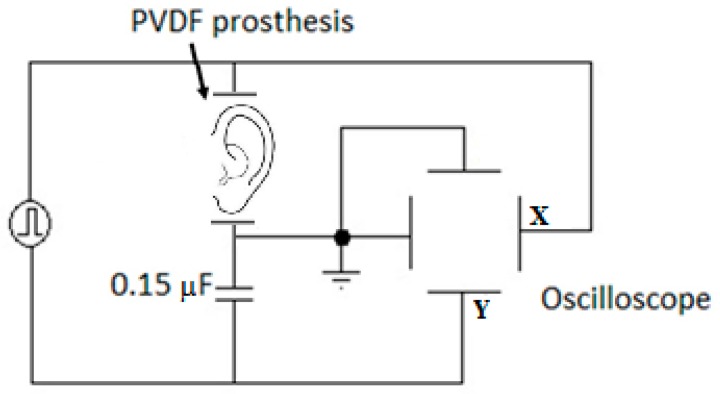
Measuring of the ferroelectric hysteresis loop using the Sawyer-Tower circuit.

In order to measure the ferroelectric hysteresis loop, the Sawyer-Tower circuit was implemented in this study (Figure 2). By measuring the voltage ($V_{L}$) across a capacitor ($C_{L}=0.15\;\mu F$) in series with the PVDF prosthesis ($C_{F}$), the charge on the ferroelectric can be determined, since: $Q_{F}=C_{L}\times V_{L}$. A sine wave was applied to the circuit with a function generator, Rigol Model DG4062; the *X* channel was measured from an *X-Y* trace using an oscilloscope, Tektronix Model MSO 3014. 

#### 2.4.2. Pressure and Temperature Characterization

**Figure 3:**
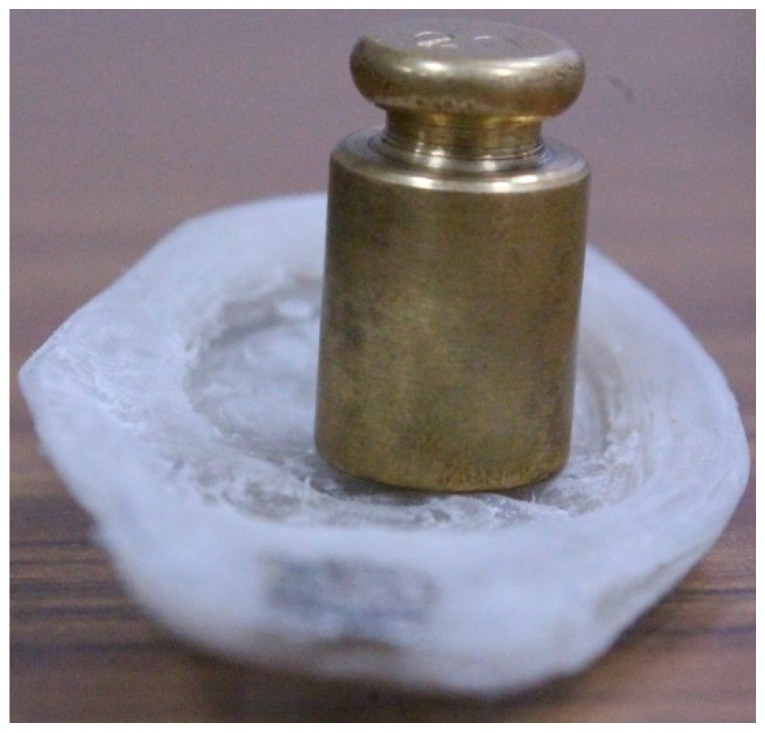
Example of pressure characterization.

The ear prosthesis of PVDF was tested for pressure measurement with different pressure loads between 0 and 16,350 Pa using a certified weight set from OHAUS. The ear prosthesis of PVDF was set in the horizontal position, and weights between 25 g and 3 kg were placed over the printed ear, as shown in Figure 3.

Regarding the temperature characterization, this has been performed by laying the ear prosthesis of PVDF in an ice bath from 5 °C to 25 °C and in a chamber furnace from 25 °C to 90 °C (in 5 °C intervals). The graphic characterization of temperature was done up to 90 °C, because the experiments we did to get the temperature response (>90 up to 150 °C) remained unchanged, which was confirmed preliminarily by Davis, who recommends the maximum temperature of operation of the PVDF to be 80 °C (melting point of around 177 °C) [10]. Moreover, for the ear prosthesis of PVDF, the temperature response is in the range of normal temperature for humans (36.19 °C (97 °F) to 37.2 °C (99 °F)). 

Regarding the property or the behavior of ear prosthesis of PVDF at low temperature under 0 °C, our research group has decided to observe the response to low temperature (<5 to −40 °C) of the sample ear prosthesis fabricated in PVDF by the 3D printer. We are implementing the equipment to do the experimental arrangement for controlling and measuring the low temperature; the results will be the subject of another publication. In this context, as reported by the manufacturers, the working limit at low temperature of PVDF is −35 °C, so the research question arises: What is the response at low temperature of the ear prosthesis made of PVDF? We will obtain the answer once that instrumentation is properly installed and calibrated. Even this is reported in the literature, and technical notes about the PVDF behavior at low temperatures indicate that the PVDF has a glass transition temperature (Tg) of about −35 °C and is typically 50% to 60% crystalline, with the maximum low temperature operation being −50 °C (Hylar^®^ Kynar^®^).

Moreover, the PVDF can be used at temperatures of −80 to 300 °F (−62 to 149 °C). The polyvinylidene fluoride (PVDF) is a fluorocarbon classified as “self-extinguishing, Group 1” (Underwriters Laboratories, Inc., Northbrook, IL, USA). It is not affected by prolonged exposure to sunlight or other sources of ultraviolet radiation. It retains its properties under high vacuum and gamma radiation and also is resistant to most acids and alkalis (Porex Corporation, Fairburn, GA, USA). Additionally, the company Bove-ag indicates the following limits of the working temperature for PVDF: A 150 °C upper limit and a lower limit of −30 °C. Additionally, Goodfellow reports the thermal properties of PVDF, with a lower working temperature of −40 °C and an upper working temperature of 135 to 150 °C.

**Figure 4:**
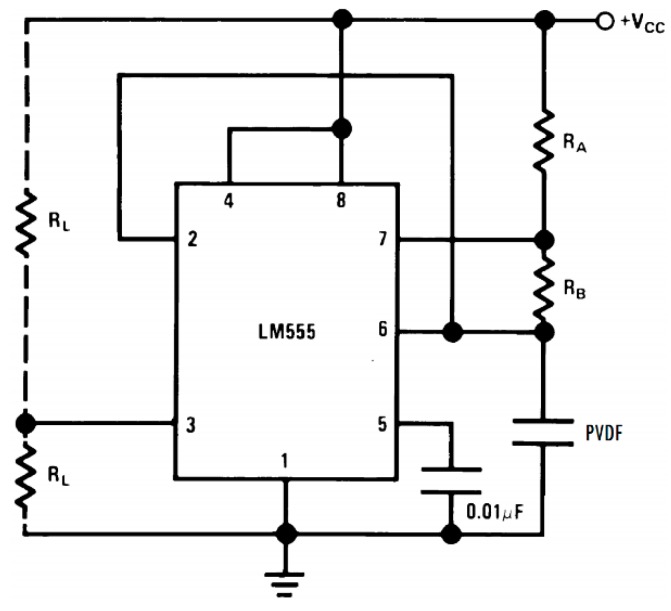
Astable multivibrator circuit LM555 to register the changes of the temperature and pressure of the PVDF prosthesis.

The basic circuit used for detecting pressure and temperature in the ear prosthesis of PVDF is displayed in Figure 4. The circuit is a relaxation oscillator (Astable Circuit Operation LM555, Texas Instrument, Dallas, TX, USA) that alters the response or the output frequency to changes in the dielectric response capacity or PVDF, connecting Terminals 2 and 6 of the LM555 to the PVDF contact and the other contact of the PVDF to GND. This gives as a result the change of the output frequency (*f*) of the circuit of Figure 4 to detect the pressure changes and temperature of the PVDF according to the oscillator frequency, as shown in Equation (1), where C is the PVDF in the LM555.

(1) $$f=\frac{1.44}{\left(RA+2RB\right)C}\;$$

We have also used the circuit of Figure 4 as a moisture sensor [8]. Moreover, the measures of pressure and temperature were made on samples of this material PVDF (FV307910) noting that if they change their dimension area and/or distance, the answers and the trends are similar. This is clearly explained by the equation:

(2) $$C=\frac{\varepsilon_{r}\varepsilon_{0}A}{d}$$

where C is the capacitance, ε_r_ is the relative permittivity of the dielectric, ε_0_ is the vacuum permittivity (ε_0_ = 8.854 × 10^−12^ F/m), *A* is the area of the PVDF or capacitor plates and *d* is the PVDF thickness or the distance between the electrodes or plates [11].

Equation (2) describes the relationship of permittivity, capacity and the physical dimensions of the ferroelectric PVDF; when PVDF is exposed to an exerted pressure (see the standard weights, Figure 3) or when it is deformed [6].

The thermal behavior of PVDF at the dielectric response is described extensively by Jafer *et al.*, Casar *et al.* and Jia *et al.* [11,12,13].

Finally, using Equations (1) and (2), the frequency of the circuit for the astable operation of the LM555 where the PVDF is connected in the position of C is:

(3) $$f=\frac{1.44d}{\varepsilon_{r}\varepsilon_{0}A\left(R_{A}+2R_{B}\right)}$$

Demonstrating that any alteration of the PVDF in its intrinsic permittivity and/or physical dimensions changes *f* by the action of pressure and temperature in the ear made with PVDF.

## 3. Results

**Figure 5:**
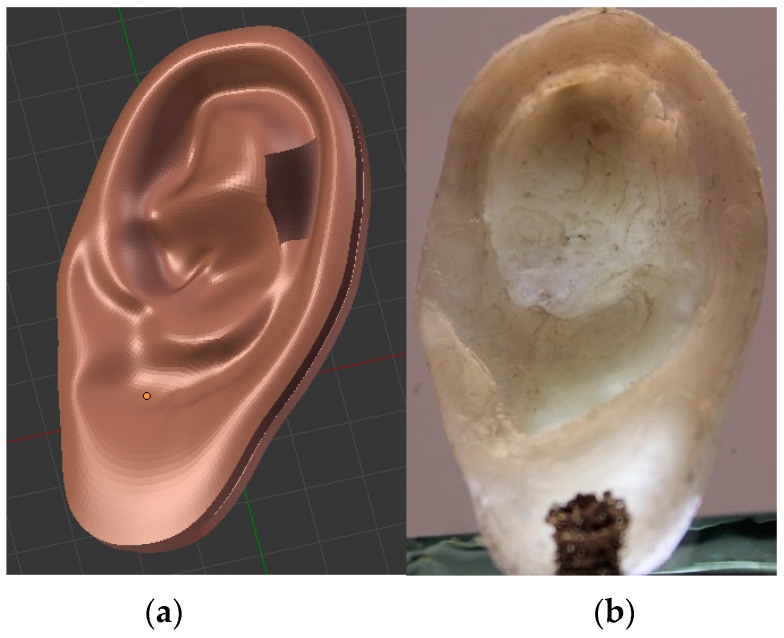
(**a**) Human ear created with a 3D CAD program. (**b**) Ear prosthesis printed from PVDF.

Figure 5a shows the design of a 3D human ear model according to the anthropometric parameters [14,15,16] and created with a computer aided design (CAD) software. It was exported as a stereolithography file in order to print it using a 3D printer.

**Figure 6:**
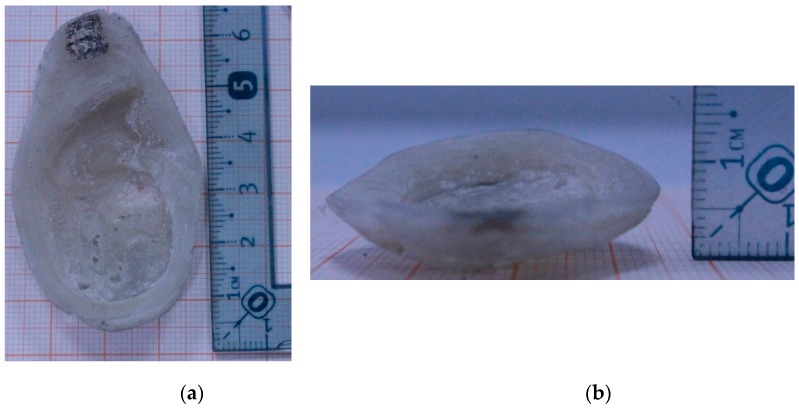
Dimensions of the ear prosthesis printed from PVDF. (**a**) Top view of the prosthesis, (**b**) Side view of the prosthesis.

The ear was printed in polyvinylidene fluoride, as it exhibits ferroelectric properties. Figure 5b shows the 3D-printed ear prosthesis. The dimensions of the printed ear are 60.1 mm wide by 34.74 mm long, and it has an average thickness of 6.88 mm. Two square electrodes of a side of 6.26 mm were painted over the printed ear with silver paint (mark SPI-Supplies). The electrodes are 41 mm apart (Figure 6). The dimensions were measured with a Starrett Vernier with a precision of 0.02 mm.

**Figure 7:**
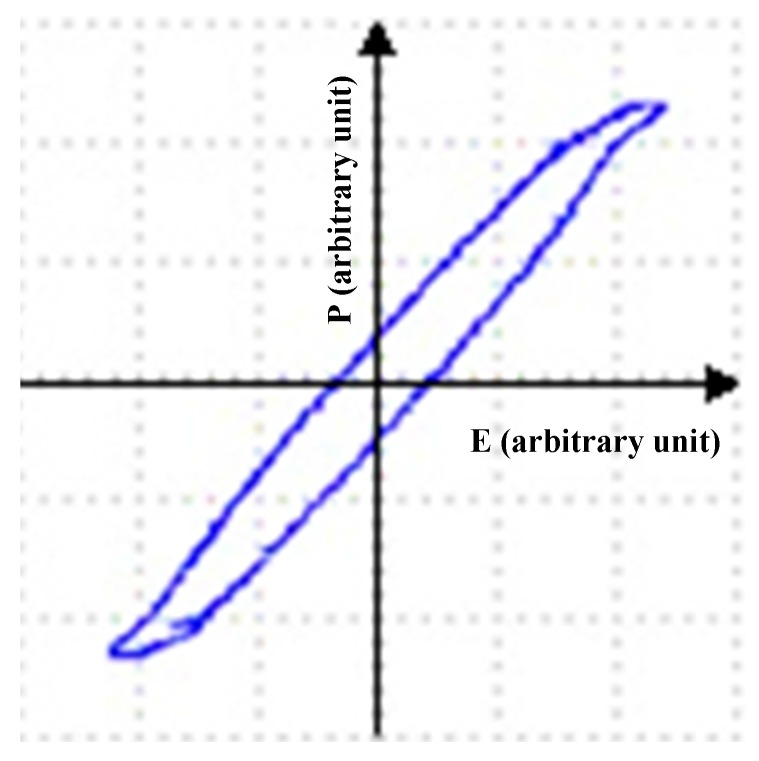
Printed PVDF hysteresis loop.

The Sawyer-Tower circuit (Figure 2) was used to measure the ferroelectric properties, such as hysteresis. Figure 7 shows the P-E hysteresis loop of the printed PVDF; the *X*-axis shows the electric field (E) in kV/cm and the *Y*-axis polarization (P) in μC/cm^2^; where P and E are arbitrary units.

The ear prosthesis of PVDF was also tested for pressure measurement applying pressure loads between 0 and 16,350 Pa. The test was carried out for both ear prostheses of PVDF (poled and unpoled).

**Figure 8:**
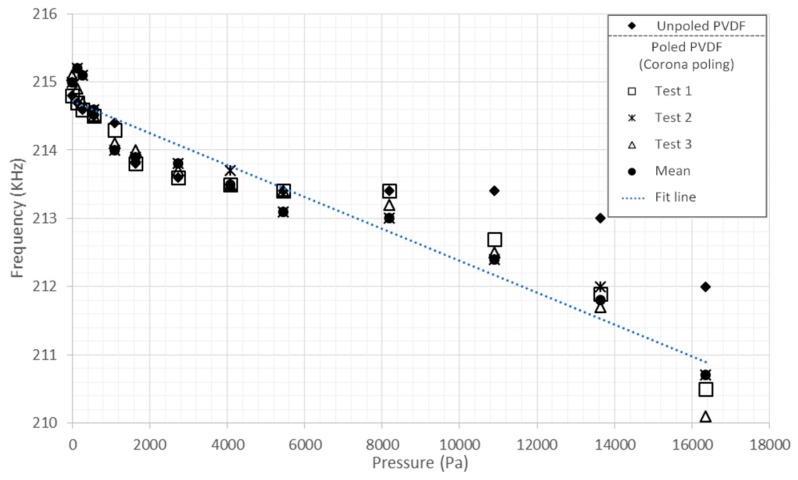
Response of the prosthesis of PVDF as a pressure sensor from 0 to 16,350 Pa.

**Figure 9:**
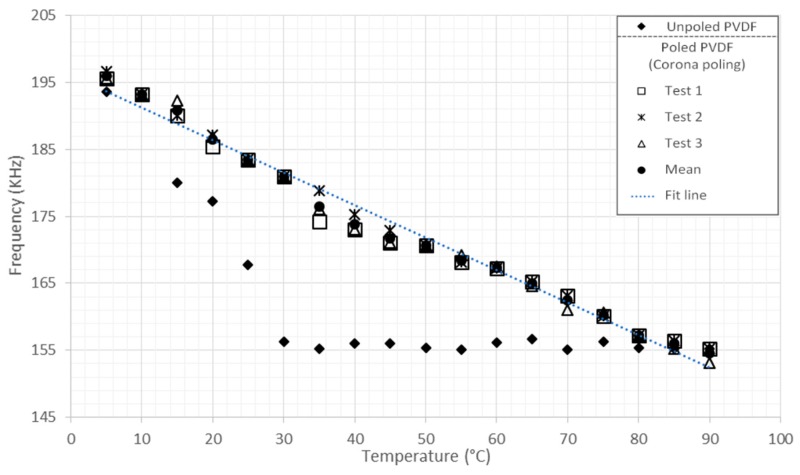
Thermal response of prostheses made of PVDF from 2 °C to 90 °C.

Regarding the characterization, we have reached the ß-phase, and this fact is clearly illustrated in Figure 8 and Figure 9, where the graphs show the differences between the unpoled material (PVDF) and the three characterizations of the same poled PVDF (Tests 1, 2 and 3); the average of these three characterizations (mean) and the line adjustment (fit line). Figure 8 shows the response of the ear prosthesis of PVDF as a pressure sensor using Equation (3).

Equation (4) fits a line through the points at 0 Pa to 16,350 Pa for the poled PVDF prosthesis with a correlation coefficient of 0.9670.

(4) y = −0.4687x + 217.16

Temperature characterization of the ear prosthesis of PVDF has been performed from 5 °C to 90 °C in 5 °C intervals. Figure 9 illustrates the response of the ear prosthesis of PVDF as a temperature sensor using Equation (3).

Equation (5) fits a line through the points at 5 °C and 90 °C for the poled PVDF prosthesis with a correlation coefficient of 0.9940.

(5) y= −2.5132x + 199.34

## 4. Discussion

The fabrication of an ear prosthesis at present has led to restoring the functionality of the middle or inner ear with an electronic hearing aid or implant, respectively [17,18,19,20,21,22], when the patient suffers a dysfunction in the mentioned parts of the ear. On the other side, the ear prosthesis is focused on two main aspects: aesthetics and functionality [23,24,25]. In recent times, mechanical and electronic prostheses that have been designed by a separate, but active prosthesis, which covers the aesthetics and functionality to restore hearing in cases where the external, middle and even the inner ear are damaged have not been developed so deeply yet [26]. 

In the case of the prosthesis as a temperature sensor, it was observed that it works linearly between 5 °C and 90 °C; after this temperature, there are no more variations. The latter consideration has no impact on the use of the prosthesis, as the ambient temperature does not exceed these ranges, and the direct application of a higher temperature may cause deformation. In addition, it has also been reported that the optimal working temperature range is from −40 °C to 100 °C. Davis reported that the maximum working temperature of PVDF is 80 °C [10].

The experimental results show an almost linear and inversely proportional behavior between the stimuli of pressure (Figure 8) and temperature (Figure 9) with the frequency response. The repeatability of the results allows one to evaluate the PVDF as a reliable material, because each stimulus applied (pressure and temperature) was tested in triplicate, getting results with slight variations, but maintaining the same trend. The prosthesis as a pressure sensor showed effectiveness in the range of 0 to 16,350 Pa without suffering any deformation, besides, it has been reported that human skin can also resist these range of pressure [27,28].

Biocompatibility is one of the subjects that also must be covered by a prosthesis, because it is permanently in contact with the skin or any other organ. That is why a PVDF prosthesis is ideal to avoid possible risks linked to hazardous substances that could affect a patient using a prosthetic device. PVDF has been used in many other kinds of biocompatible applications and has been widely studied as a safety material in biomedical applications [29,30].

Moreover, 3D printing is a technology with many benefits compared to traditional 3D manufacturing methods, such as injection molding, machining and forming. 3D printing allows one to change some aspects in the design easily. This is an important advantage in hearing aids’ manufacturing due to the fact that ears grow as we age, so changes in a patient’s prosthesis could be made quickly and easily. Although injection molding was the preferred process for manufacturing auricular prosthesis [31], 3D printing technology offers low cost and the possibility of customizing and personalizing prostheses in a fast and easy way.

Our finding could also contribute to the 3D printing of composite tissues [32] due to the biocompatibility and degradability of PVDF; so, it may be used as a scaffold. As PVDF is a highly non-reactive polymer, it has no effect on the formation and behavior of the cells used.

Table 2 is a comparative analysis of some materials used for the fabrication of ear prostheses, highlighting PVDF properties as a sensor due to its piezoelectric properties.

Although the 3D-printed ear prosthesis made of PVDF does not present the same morphology of PVDF rods due to the parameters set in Table 1, such as infill and number of shells (parameters related to density and porosity), the piezoelectric and pyroelectric properties still remain the same. Finally, by using a 3D printer, an ear prosthesis prototype could be fabricated just in a few hours with all of the advantages mentioned above.

## 5. Conclusions

In this work, a prosthesis made of PVDF was manufactured satisfactorily with a 3D printer. It was also tested as pressure and temperature sensors. The characterization could be achieved satisfactorily. As shown in Figure 8 and Figure 9, the typical response of the PVDF pressure and temperature sensors was found to be very reliable. It was seen that PVDF displayed a high sensitivity to pressure changes in the range of 0 to 16,350 Pa.

Smart PVDF prostheses provide a promising tool for measuring pressure and temperature variations due to their ferroelectric properties (piezoelectricity and pyroelectricity) [5,33]. These kinds of smart prostheses have great potentialities in the biomedical engineering field because of their ability to generate an electrical potential in response to applied mechanical stress or variations of temperature, as well as flexibility [34].

A remarkable point is that our ear prosthesis fabricated in PVDF by a 3D printer has a large potentiality to respond to sound as humans do, due to the piezoelectric effect, while the 3D-printed bionic ear of Mannoor responds to electromagnetic signals, which are different from the way in which human ear works.

Finally, it is possible to manufacture tactile sensors based on PVDF with their respective feedback, *i.e.*, the prosthesis could provide proportionate electrical stimulation to the nervous system of the skin.

## Figures and Tables

**Table 1:** Printing parameters used for PVDF.

Extruder Temperature	170 °C
Raft temperature	190 °C
Printing speed	15 mm^3^/s
Infill	40%
Number of shells	3

**Table 2:** Comparative analysis of materials used in prosthetics.

Material	Properties
PVDF	Fairly flexible Not toxic Withstands temperatures up to 90 °C *Used as a temperature sensor* *Used as a pressure sensor* Humidity resistant If adjustments are necessary, they are performed only by software
Silicon	Totally flexible Not toxic Withstands temperatures up to 250 °C It is not a sensor Humidity resistant If adjustments are necessary, you have to make a new mold
Composite Tissue	Not flexible Not toxic Withstands temperatures up to 80 °C It is not a sensor If adjustments are necessary, they are performed only by software
